# Deer antler peptides attenuate inflammation, oxidative stress, and pyroptosis in *Salmonella*-infected macrophages and murine colonic tissues

**DOI:** 10.5713/ab.260612

**Published:** 2026-09-01

**Authors:** Deshou Zeng, Haihua Xing, Zihui Sun, Yukai Ma, Qing Wang, Fuquan Yin, Zhenhua Gao, Heping Li

**Affiliations:** 1 College of Wildlife and Protected Area, Northeast Forestry University, Harbin, China; 2 College of Coastal Agriculture Science, Guangdong Ocean University, Zhanjiang, China

**Keywords:** Deer Antler Peptides, Inflammatory Response, NRF2, Oxidative Stress, Pyroptosis, *Salmonella*

## Abstract

**Objective:**

Deer antler peptides (DAPs) are well documented for their potent anti-inflammatory and antioxidant properties, although their efficacy in alleviating pathogen-induced intestinal infections mechanisms remain poorly understood. In this study, we investigated the protective effects of DAPs against *Salmonella*-induced intestinal inflammation and associated tissue injury, while elucidating the molecular mechanisms involved.

**Methods:**

Primary peritoneal macrophages isolated from wild-type (WT) and NRF2 knockout (*nrf2*^−/−^) mice were infected with *Salmonella*; concurrently, we established a murine model of *Salmonella*-induced intestinal inflammation by infecting WT and *nrf2*^−/−^ mice. Using both *in vitro* and *in vivo* systems, we evaluated the immunomodulatory effects of DAPs during *Salmonella* infection.

**Results:**

*In vitro*, the results demonstrated that DAPs significantly inhibited the *Salmonella*-induced production of TNF-α and IL-6, the activation of MAPK signaling pathway, GSDMD-mediated pyroptosis, and excessive reactive oxygen species accumulation. Mechanistically, DAPs activate the NRF2 signaling pathway by promoting nuclear translocation and the transcriptional upregulation of HO-1 and NQO-1. The genetic deletion of NRF2 substantially attenuated the protective effects of DAPs against inflammation, pyroptosis, and oxidative stress, thus confirming the dependence of this mechanism on this pathway. *In vivo*, DAPs significantly reduced intestinal histopathological changes, epithelial barrier disruption, pyroptosis, and oxidative damage in WT mice, however these protective effects were markedly diminished in *nrf2*^−/−^ mice. Notably, DAPs significantly reduced colonic bacterial loads in both WT and *nrf2*^−/−^ mice while concurrently suppressing pyroptosis. Our findings indicate that DAPs may exert antibacterial effects through a mechanism independent of both NRF2 activation and the NLRP3-mediated pyroptotic pathway; however, the underlying molecular mechanism remains to be fully elucidated.

**Conclusion:**

Our findings reveal DAPs function as multi-modal regulators that coordinately suppress inflammation, pyroptosis, and oxidative stress through NRF2 activation during *Salmonella* infection, and provides mechanistic insights and a translational basis for the application of animal-derived peptides in the treatment of infectious intestinal disorders.

## INTRODUCTION

As one of the leading zoonotic foodborne pathogens worldwide [1], *Salmonella*, a Gram-negative, facultative anaerobic bacillus belonging to the Enterobacteriaceae family within the order Enterobacter [2], exhibits broad host tropism across livestock species, including swine, chickens, and ducks, causing acute gastroenteritis, septicemia, and systemic typhoidal disease [3]. Annually, *Salmonella* infection accounts for approximately 93.8 million cases of gastroenteritis worldwide, leading to an estimated 155,000 fatalities. In Korea, 544 cases of confirmed gastroenteritis outbreaks were reported in 2017 [4]. In neonatal and juvenile animals, *Salmonella* infection often leads to high morbidity and mortality, imposing substantial economic burdens on the livestock industry. Critically, certain serovars undergo vertical transmission in avian breeding flocks [5], enabling the persistent colonization of reproductive tracts and subsequent egg contamination. Once established in a production facility, such infections are notoriously difficult to eradicate, posing persistent biosecurity risks not only to the affected farm but also to neighboring operations through environmental shedding and transmission via personnel or vehicles, thereby threatening the sustainability of regional livestock health [6]. Meanwhile, the rising global demand for animal-derived foods, such as meat, eggs, and dairy, has increased human exposure to contaminated products [7]. Through the food chain, *Salmonella* can cause a range of clinical manifestations, from mild gastroenteritis to severe typhoid fever, enteric fever, and even sepsis, seriously endangering the security of public health [8]. According to data on the global burden of disease data from the World Health Organization (WHO), there were 95.2 million new cases of *Salmonella* infection in 2017, with 50,771 associated deaths [9].While current control strategies rely primarily on vaccination and antimicrobial therapy, both approaches face significant limitations. Licensed vaccines provide incomplete protection and fail to prevent asymptomatic carriage or environmental persistence [10], while routine subtherapeutic antibiotic use in livestock disrupts commensal gut microbiota, impairs mucosal immunity, and exerts strong selective pressure that accelerates the emergence of multidrug-resistant and extensively drug-resistant *Salmonella* strains [11]. Therefore, there is an urgent need for novel, non-antibiotic intervention strategies that combine precise pathogen targeting, favorable safety profiles, and direct antimicrobial activity with host-directed immunomodulation and intestinal barrier stabilization to safeguard livestock gut health and advance the transition toward sustainable, antibiotic-free livestock production.

*Salmonella* infection triggers a robust host immune response characterized by the rapid generation of reactive oxygen species (ROS) in phagocytic cells, particularly in neutrophils and macrophages [12]. At physiological concentrations, ROS act as critical antimicrobial effectors by inducing oxidative damage to bacterial DNA, proteins, and membranes, thereby playing an essential role in intracellular pathogen clearance [13]. However, dysregulated or excessive ROS production causes collateral oxidative injury to host macromolecules, including nuclear and mitochondrial DNA, functional and structural proteins, and polyunsaturated membrane lipids, resulting in epithelial and immune cell dysfunction, disruption of the intestinal epithelial barrier, and amplification of mucosal inflammation [14]. Mechanistically, elevated ROS levels promote the dissociation of thioredoxin-interacting protein (TXNIP) from reduced thioredoxin, freeing TXNIP to bind and activate the NLRP3 inflammasome, a central signaling complex that drives Caspase-1-dependent pyroptosis [15]. Consequently, ROS serve as a redox-sensitive second messenger that functionally couples oxidative stress with inflammasome activation. While controlled pyroptosis facilitates *Salmonella* containment via the lytic release of pro-inflammatory cytokines and danger-associated molecular patterns (DAMPs), a pathological hyperactivation of this pathway impairs macrophage viability, facilitates intracellular bacterial escape, promotes systemic dissemination, and worsens disease severity [16]. Collectively, the concurrent suppression of *Salmonella*-induced oxidative stress and NLRP3-mediated pyroptosis represents a mechanistically grounded and therapeutically promising strategy for alleviating intestinal inflammatory pathology.

Deer antlers, typically harvested once or twice annually, represent the only mammalian organ capable of complete, annual regeneration [17]. Following their removal, wound healing at the pedicle proceeds rapidly with minimal intervention, which can include topical applications of plant-derived ash to achieve hemostasis, providing direct evidence for the presence of endogenous wound-healing factors within antler tissue [18]. Notably, post-harvest infections are exceptionally rare in deer, suggesting that antler tissue may contain intrinsic antimicrobial agents. Because of this, deer antler peptides (DAPs) identified as the principal bioactive components of antlers, have attracted growing scientific interest. Accumulating evidence has indicated that DAPs exert diverse pharmacological effects, including immunomodulation [19], anti-osteoporotic activity [20], antitumor effects [21], anti-inflammatory properties [22], and a mitigation of oxidative stress [23]. Although a prior study has explored the antibacterial activity of deer antler extracts against pathogenic microorganisms [24], the specific molecular mechanisms underlying DAPs-mediated antibacterial action remain largely undefined. Accordingly, in this study, we investigate whether naturally derived DAPs alleviate *Salmonella*-induced intestinal inflammation, oxidative stress, and pyroptosis, and we further elucidate the molecular mechanisms of this process.

## MATERIALS AND METHODS

### Bacterial culture and infection

*Salmonella* strain SL1344 (Cat#bio-110527; Biobw) was cultured in Luria–Bertani broth at 30°C. For cell infection assays, macrophages were pre-treated with DAPs for 12 h and subsequently infected with Salmonella at a multiplicity of infection (MOI) of 10 for 1 h.

### Deer antler peptides extraction

DAPs were extracted under the supervision of Professor Zhen Liu (Qingdao Agricultural University). The extraction steps proceeded as follows: Fresh deer antler was washed with sterile normal saline to remove surface blood, fine velvet hair, and adherent debris, followed by brief disinfection with 75% (v/v) ethanol. The cleaned antler was then sliced into 0.5 cm thick sections to ensure consistent particle size during downstream processing. To prepare the tissue homogenates, the slices were immersed in cold normal saline (4°C) and mechanically disrupted using a high-speed tissue grinder until a homogeneous suspension was obtained. Ultrasonic disruption was subsequently performed on ice to further disrupt cellular membranes and enhance intracellular peptide release. Enzymatic hydrolysis was conducted in two sequential steps: first, neutral protease was added to the homogenate, and incubated at 37°C and pH 7.0 for 5 h; second, the pH was adjusted to 8.0, and alkaline protease and papain were added, followed by incubation at 55°C for 6 h. Enzyme inactivation was achieved by adjusting the pH to 9.0. The resulting digest was centrifuged at 5,000×g for 15 min at 4°C to pellet insoluble residues, and the supernatant was carefully collected. Finally, the supernatant was subjected to vacuum freeze-drying to yield a stable, bioactive DAPs powder. The final DAPs preparation exhibited a purity of 90.91%, satisfying the stringent purity criteria required for functional assays.

### Peritoneal primary macrophage isolation and culture

C57BL/6 WT and *nrf2*^−/−^ mice, aged 6–8 weeks, were intraperitoneally injected with 3 mL of sterile thioglycollate broth. Mice were housed under standard pathogen-free conditions for 72 h post-injection. Euthanasia was performed by cervical dislocation; immediately thereafter, the abdominal surface was disinfected with 75% (v/v) ethanol. The abdominal skin was incised, and the exposed peritoneal surface was thoroughly wiped with a 75% ethanol-soaked cotton swab. Subsequently, 5 mL of ice-cold normal saline was injected into the peritoneal cavity, and the abdomen was gently massaged for 1 min to facilitate cell detachment and suspension. Peritoneal lavage fluid was collected and centrifuged at 300×g for 5 min at 4°C, and the supernatant was carefully discarded. The resulting cell pellet was resuspended in complete primary macrophage culture medium. Cells were seeded into a culture dish and incubated at 37°C with 5% CO_2_ for 3 h to allow adherence. Non-adherent cells were removed by gentle washing with normal saline, and the adherent population was confirmed as primary peritoneal macrophages.

### CCK-8 assay

Primary peritoneal macrophages were seeded into 96-well culture plates at a density of 1×10^5^ cells/well and incubated overnight at 37°C in a humidified 5% CO_2_ incubator. The following day, the culture medium was replaced with fresh complete macrophage medium supplemented with serially diluted DAPs at final concentrations of 0, 5, 10, 15, 20, 25, 30, 35, 40, and 50 mg/mL. The cells were exposed for 12 h, with six biological replicates per concentration. Thereafter, 10 μL of CCK-8 reagent (Solarbio) was added to each well, and plates were incubated for an additional 30 min under the same conditions (37°C, 5% CO_2_). Absorbance at 450 nm was measured using a microplate reader, following the manufacturer’s protocol.

### Quantitative reverse transcription polymerase chain reaction

Total RNA was extracted from cultured cells and colonic tissues using a commercial RNA extraction kit (Yeasen), in accordance with the manufacturer’s protocol. RNA concentration and purity were assessed using a NanoDrop (Thermo Fisher Scientific), and cDNA synthesis was performed using 1 μg of total RNA and a commercial reverse transcription kit (Yeasen). Quantitative reverse transcription polymerase chain reaction (qRT-PCR) was conducted using SYBR Green Master Mix (Yeasen) on a Bio-Rad Real-Time PCR Detection System. Reactions were carried out under the following cycling conditions: initial denaturation at 95°C for 5 min, followed by 40 cycles of 95°C for 10 s and 60°C for 30 s. All primer sequences were synthesized by Sangon Biotech and are listed below: TNF-α, F: 5′-TAGCCCACGTCGTAGCAAAC-3′, R: 5′-TGTCTTTGAGATCCATGCCGT-3′; IL-6, F: 5′-GGGACTGATGCTGGTGACAA-3′, R: 5′-ACAGGTCTG TTGGGAGTGGT-3′; GAPDH, F: 5′-GGGGTCGTTG ATGGCAACA-3′, R: 5′-AGGTCGGTGTGAACGGA TTTG-3′. Relative mRNA expression levels were calculated using the 2^ΔΔCt^ method, with GAPDH serving as the endogenous reference gene for normalization.

### Enzyme-linked immunosorbent assay

Cell culture supernatants from macrophages and homogenates of colonic tissue were prepared in ice-cold RIPA lysis buffer. Standard curves were generated using serial dilutions of recombinant cytokine standards. To prepare these, 100 μL aliquots of samples or diluted standards were added to pre-coated 96-well plates and incubated at 37°C for 90 min. Plates were washed three times with phosphate-buffered saline (PBS). Subsequently, 100 μL of biotinylated detection antibody was added to each well and incubated at 37°C for 50 min. After additional PBS washes, 100 μL of horseradish peroxidase (HRP)-conjugated streptavidin was added, and plates were incubated for 50 min at 37°C. Thereafter, 90 μL of TMB substrate solution was added to each well, and plates were incubated in the dark at 37°C for 20 min. The enzymatic reaction was terminated by adding 50 μL of stop solution. Optical density (OD) was measured at 450 nm using a microplate reader, and cytokine concentrations were interpolated from the respective standard curves. Enzyme-linked immunosorbent assay (ELISA) kits for TNF-α, IL-6, IL-1β and IL-18 were obtained from ELK Biotechnology.

### Western blot

Nuclear protein extraction was performed using the Ultra Nuclear Protein Extraction Kit (Solarbio, Cat#: EX1471) following the manufacturer’s protocol. Briefly, upon completion of *Salmonella* infection, cell suspensions were centrifuged at 500×g for 5 min at 4°C, the supernatant culture medium was carefully aspirated, and cell pellets were collected. To achieve nuclear cytoplasmic fractionation, 2×10^6^ cells were resuspended in 200 μL of ice-cold Reagent A, gently pipetted to achieve homogeneous mixing, and incubated with constant shaking for 20 min at 4°C, after which the mixture was centrifuged at 13,000×g for 5 min at 4°C, and the resulting supernatant was harvested as the cytoplasmic protein fraction. The remaining crude nuclear pellet was resuspended in PBS and centrifuged at 5,000×g for 5 min at 4°C; the supernatant was discarded to remove residual cytoplasmic contaminants, and the purified nuclear pellet was then resuspended in 100 μL of ice-cold Reagent B, vigorously vortexed for 15 s, and incubated with shaking for 30 min at 4°C. Finally, the mixture was centrifuged at 12,000×g for 10 min at 4°C, and the resulting supernatant was collected as the nuclear protein fraction.

Cells or colonic tissues were homogenized in ice-cold RIPA lysis buffer containing 1 mM PMSF (Solarbio) and a 1% protease and phosphatase inhibitor cocktail (Solarbio). Total protein concentration was quantified using the BCA Protein Assay Kit (Thermo Fisher Scientific) following the manufacturer’s protocol. Equal amounts of protein lysate were subjected to SDS-PAGE on 10% polyacrylamide gels and subsequently electrotransferred onto PVDF membranes. Membranes were blocked for 2 h at room temperature with 5% (w/v) non-fat milk in TBST, followed by overnight incubation at 4°C with primary antibodies against COX-2 (ABclonal), NRF2 (ABclonal), HO-1 (ABclonal), NQO1 (Zenbio), p-ERK (Zenbio), ERK (Zenbio), p-JNK (ABclonal), JNK (ABclonal), p-p38 (ABclonal), p38 (ABclonal), NLRP3 (Zenbio), GSDMD-NT (ABclonal), Caspase-1 (Zenbio), and β-actin (Zenbio). After washes with TBST, membranes were incubated with HRP-conjugated secondary antibody (Zenbio) for 1 h at room temperature. Immunoreactive bands were visualized using enhanced chemiluminescence (ECL) detection reagent (Yeasen) and captured with a chemiluminescence imaging system.

### Hoechst/reactive oxygen species staining

Primary peritoneal macrophages were seeded into 12-well plates at a density of 1×10^6^ cells per well. Following adherence for 4–6 h under standard culture conditions (37°C, 5% CO_2_), cells were pre-treated with DAPs for 12 h. Subsequently, macrophages were infected with *Salmonella* (SL1344) at a MOI of 10 for 1 h. After infection, extracellular bacteria were removed by gentle washing with ice-cold PBS. Cells were then incubated in serum-free DMEM containing 10 μM DCFH-DA (Yeasen) and 5 μg/mL Hoechst 33342 (Yeasen) for 30 min at 37°C in the dark. Unincorporated fluorescent probes were removed through two additional washes with ice-cold PBS, and cellular fluorescence was visualized using a fluorescence microscope.

### Hoechst/propidium iodide staining

Primary peritoneal macrophages were seeded into 24-well plates at a density of 5×10^5^ cells per well. Following adherence for 4–6 h under standard culture conditions (37°C, 5% CO_2_), the cells were pre-treated with DAPs for 12 h, and subsequently infected with *Salmonella* at an MOI of 10 for 1 h. Extracellular bacteria were removed through gentle washing with ice-cold PBS. The cells were then incubated in serum-free DMEM containing 5 μg/mL Hoechst 33342 (Yeasen) and 5 μg/mL propidium iodide (PI) (Yeasen) for 30 min at 37°C in the dark. Unbound fluorescent dyes were removed by washing with ice-cold PBS, and cells were observed and imaged using a fluorescence microscope.

### Malondialdehyde/glutathione/superoxide dismutase measurement

Primary peritoneal macrophages (5×10^6^ cells per well) were seeded into 6-well plates. Following adherence for 4–6 h under standard culture conditions (37°C, 5% CO_2_), cells were pre-treated with DAPs for 12 h and subsequently challenged with *Salmonella* at an MOI of 10 for 1 h. Extracellular bacteria were removed through gentle washing with ice-cold PBS. Cells were harvested on ice using cold PBS, lysed by sonication, and centrifuged at 12,000×g for 15 min at 4°C to obtain supernatants. The content of malondialdehyde (MDA) (Solarbio) and glutathione (GSH) (Solarbio), as well as superoxide dismutase (SOD) (Solarbio) activity, were quantified using respective commercial assay kits according to the manufacturers’ instructions.

### Mouse model

Male C57BL/6 WT and NRF2 knockout (*nrf2*^−/−^) mice, aged 6–8 weeks, were purchased from Jiangsu Cyagen. Mice were housed in a specific pathogen-free facility at Guangdong Ocean University under controlled conditions, with a temperature of 22°C–23°C and a 12-h light/dark cycle. Standard rodent chow and autoclaved tap water were provided *ad libitum*. This study included 6 groups: WT control, WT–*Salmonella*, WT–*Salmonella*+DAPs, *nrf2*^−/−^ control, *nrf2*^−/−^–*Salmonella*, and *nrf2*^−/−^–*Salmonella*+DAPs. Each group included 8–10 C57BL/6 mice. The *Salmonella*+DAPs groups received 50 mg/kg DAPs (dissolved in normal saline) via daily intragastric gavage for 7 consecutive days, while the control groups received an equivalent volume of normal saline. Next, the *Salmonella* and *Salmonella*+DAPs groups received 20 mg streptomycin in drinking water to suppress intestinal commensal microbiota; 24 h later, they were orally inoculated with 1×10^7^ CFU of *Salmonella* SL1344 per mouse. Mice were humanely euthanized 72 h post-infection, and colonic tissues were collected for downstream molecular and histopathological analyses.

### Histological analysis and disease activity index

Tissue sections were stained with hematoxylin and eosin (H&E) using an H&E Stain Kit (Solarbio), according to the manufacturer’s protocols. Briefly, paraffin-embedded sections were dewaxed in xylene and rehydrated stepwise through a graded ethanol series. Sections were stained with hematoxylin for 2 min, differentiated in 1% hydrochloric acid-ethanol for 5–10 s until nuclear chromatin exhibited clear blue staining, rinsed thoroughly with distilled water, and incubated in 1% ammonium hydroxide for 30 s to enhance blue contrast. Following counterstaining with eosin for 1 min, sections were dehydrated through a reverse graded ethanol series and sealed with neutral balsam. Histological images were observed and imaged using a microscope. Histological scores were assessed based on two parameters: epithelial damage and inflammatory cell infiltration. Epithelial damage scoring was as follows: 0 = normal morphology; 1 = focal goblet cell loss; 2 = extensive goblet cell loss; 3 = focal crypt loss; 4 = extensive crypt loss. Inflammatory cell infiltration was measured as follows: 0 = no infiltration; 1 = infiltration limited to crypt bases; 2 = infiltration extending to the muscularis mucosa; 3 = extensive infiltration of the muscularis mucosa with marked edema; 4 = infiltration spreading to the submucosa. Disease activity index (DAI) scores were assessed based on three parameters: weight loss, stool consistency, and fecal blood. Weight loss scoring was as follows: 0 = no weight loss; 1 = 1%–5% weight loss; 2 = 6%–10% weight loss; 3 = 11%–18% weight loss; 4 = more than 18% weight loss. Stool consistency scoring was as follows: 0 = normal formed stool; 1 = soft but formed stool; 2 = soft, loose stool; 3 = extremely soft, watery stool; 4 = watery diarrhea. Fecal blood was measured as follows: 0 = no visible blood; 1 and 2 = focal bloody feces; 3 = trace blood in stool; 4 = severe rectal bleeding. All histopathological and DAI assessments were conducted by blinded observers, and the scoring criteria were adapted from a previously published study [25].

### Colony-forming units

Mouse colon tissues were harvested and immersed in streptomycin sulfate solution for 30 min. Next, tissues were retrieved, the surface residual solution was carefully removed via sterile absorbent paper, and individual tissue weights were recorded. Sterile normal saline was added at a standardized ratio of 1:3 (tissue weight: saline volume) to each sample, and tissue homogenates were prepared via mechanical disruption. Homogenates were then serially diluted to generate 10^–3^ and 10^–4^ dilution gradients, with aliquots of each dilution evenly spread onto duplicate SS selective agar plates. Plates were incubated at 37°C under aerobic conditions overnight, and CFUs were enumerated 12 h post-incubation. The final results are presented as CFU/mg of colon tissue.

### Immunohistochemical staining

Immunohistochemistry (IHC) was performed using a commercial IHC staining kit (Boster), following the manufacturer’s instructions. Briefly, sections were deparaffinized in xylene, rehydrated through graded ethanol, subjected to heat-induced antigen retrieval in 10 mM sodium citrate buffer via microwave heating (95°C for 15 min), and treated with 3% hydrogen peroxide for 15 min to quench endogenous peroxidase activity. Sections were blocked with 5% bovine serum albumin (BSA) in PBS for 1 h at room temperature, followed by overnight incubation at 4°C with primary antibody diluted in blocking buffer. The primary antibodies were MUC2 (Boster), ZO-1 (Boster), and Occludin (Boster). A biotinylated secondary antibody was applied for 1 h at room temperature. Immunoreactive signals were visualized using a 3,3′-diaminobenzidine (DAB) (Boster) substrate solution, terminated by rinsing in distilled water. Sections were counterstained with hematoxylin, differentiated in 1% hydrochloric acid-ethanol, blued in 1% ammonium hydroxide, dehydrated through ascending ethanol series, cleared in xylene, and permanently mounted with neutral balsam. Images were observed and imaged using a microscope.

### Intestinal permeability

Mice were orally gavaged with FITC-dextran (molecular weight, 3–5 kDa, Sigma-Aldrich) at a dose of 0.6 mg/kg body weight, suspended diluted in sterile saline. Four hours post-gavage, blood was harvested and kept at room temperature for 30 min for clot formation. Serum was isolated through centrifugation at 1,500×g for 15 min at 4°C. FITC-dextran fluorescence concentration in serum was detected at an excitation of 485 nm and an emission of 528 nm using a microplate reader. Sample concentrations were determined in reference to a standard curve generated from serially diluted FITC-dextran in blank serum.

### Statistical analysis

Statistical analyses were performed using GraphPad Prism 10.0, and data are presented as means±standard deviation. Multiple-group comparisons were assessed using one-way ANOVA, followed by Tukey’s multiple comparisons test, and differences were considered significant at * p<0.05, ** p<0.01, *** p<0.001 and **** p<0.0001; “ns” denotes not significant. All experiments were carried out at least three independent replicates. All *in vitro* experiments included at least three biologically independent replicates. Primary peritoneal macrophages were isolated from different mice at different time to eliminate shared biological or technical confounders. For *in vivo* experiments, each treatment group comprised 8–10 mice.

## RESULTS

### Deer antler peptides inhibit inflammatory response during *Salmonella* infection

To evaluate the immunomodulatory potential of DAPs in *Salmonella*-infected macrophages, we first assessed their cytotoxic threshold in primary murine peritoneal macrophages using the CCK-8 assay. Exposure to DAPs at concentrations ≥10 mg/mL significantly reduced cell viability, whereas 5 mg/mL exerted no cytotoxic effect relative to the untreated control (Supplement 1A). Accordingly, 5 mg/mL was selected as the non-cytotoxic working concentration for all subsequent functional experiments. Given the established evidence showing that DAPs exert potent anti-inflammatory activity in multiple sterile inflammatory models, including LPS-induced acute lung injury and DSS-induced colitis, we next investigated whether this activity extends to infection-associated inflammation. Our results demonstrated that, while *Salmonella* infection significantly upregulated TNF-α and IL-6 mRNA expression and protein secretion, pre-treatment with DAPs markedly attenuated both transcriptional induction and protein release (Figure 1A–1D). Mechanistically, Western blot analysis showed that *Salmonella* infection markedly enhanced the phosphorylation of ERK, JNK, and p38 signaling pathways, whereas pre-treatment with DAPs effectively suppressed the activation of all three kinases (Figure 1E–1H). Collectively, these findings indicate that DAPs exert anti-inflammatory effects in *Salmonella*-infected macrophages primarily through an inhibition of the MAPK signaling pathway.

### Deer antler peptides mitigate pyroptosis in *Salmonella-*infected macrophages

Next, we systematically evaluated the impact of DAPs on *Salmonella*-induced pyroptosis in primary murine peritoneal macrophages. Hoechst 33342/PI dual staining revealed that *Salmonella* infection triggered pyroptotic cell death, whereas pre-treatment with DAPs markedly attenuated the death of macrophages (Figure 2A). Consistently, DAPs significantly suppressed the *Salmonella*-induced synthesis of LDH (Figure 2B), demonstrating an anti-pyroptotic activity. Because LDH synthesis reflects general plasma membrane disruption and is not specific to pyroptosis, we further assessed canonical pyroptosis execution molecules. *Salmonella* infection strongly induced the cleavage and secretion of IL-1β and IL-18 (Figure 2C, 2D), concomitant with an upregulated protein expression of NLRP3 inflammasome components, including NLRP3, Caspase-1, and the GSDMD-NT terminal fragment (Figure 2E–2H). Collectively, these findings demonstrate that DAPs suppress *Salmonella*-induced macrophage pyroptosis by suppressing the activation of the NLRP3/Caspase-1/GSDMD signaling axis.

### Deer antler peptides suppress oxidative stress in *Salmonella*-infected macrophages

*Salmonella* infection activates a coordinated network of host defense response, including inflammation, pyroptosis, and oxidative stress. Given the well-established antioxidant properties of DAPs in diverse models, we investigated whether DAPs exert protection against *Salmonella*-induced oxidative damage in macrophages. Western blot analysis revealed that, although *Salmonella* infection significantly upregulated COX-2 expression, pre-treatment with DAPs significantly suppressed this induction (Figure 3A). Consistently, *Salmonella* infection triggered severe redox imbalance evidenced by elevated MDA levels (Figure 3B), depleted GSH (Figure 3C), and diminished SOD enzymatic activity (Figure 3D); however, all of these were significantly ameliorated by the DAPs treatment. Intracellular ROS accumulation, quantified through DCFH-DA staining, was robustly increased upon infection and was potently attenuated by DAPs treatment (Figure 3E). Collectively, these findings demonstrate that DAPs provide comprehensive antioxidant protection against *Salmonella*-induced oxidative stress. Given that NRF2 is the master transcriptional regulator of the endogenous antioxidant response, we next examined whether DAPs modulate its activation. Immunoblotting of nuclear protein demonstrated that DAPs significantly enhance NRF2 nuclear translocation (Figure 3F). Moreover, DAPs not only increased total NRF2 protein abundance but also robustly upregulated its canonical downstream target proteins, HO-1 and NQO-1 (Figure 3G–3J). Thus, DAPs alleviate *Salmonella* infection-associated oxidative injury predominantly through an activation of the NRF2/HO-1/NQO-1 signaling axis.

### Deer antler peptides attenuate oxidative stress, inflammatory responses, and pyroptosis through modulation of the NRF2 signaling pathway

Prior studies have implicated the NRF2 signaling pathway in DAPs-mediated immunomodulation. To determine whether NRF2 is functionally required for DAPs to modulate the innate immune response to *Salmonella* infection, we performed genetic loss-of-function validation using primary peritoneal macrophages isolated from *nrf2*^−/−^ mice. In NRF2-deficient macrophages, DAPs completely failed to suppress *Salmonella*-induced intracellular ROS accumulation (Figure 4A), and the ability of DAPs to attenuate MDA elevation (Figure 4B) and restore GSH levels (Figure 4C) was significantly impaired. Consistent with these observations, Western blot analysis revealed that DAPs failed to upregulate the expression of the canonical NRF2 target proteins HO-1 (Figure 4D) and NQO-1 (Figure 4E) in the absence of NRF2, suggesting that NRF2 serves as an obligate transcriptional mediator of DAPs-induced antioxidant responses. Importantly, NRF2 deficiency also abrogated the anti-inflammatory effects of DAPs, which lost their ability to inhibit TNF-α (Figure 4F) and IL-6 (Figure 4G) secretion, and failed to suppress the phosphorylation of ERK, JNK, and p38 (Figure 4H–4K) signaling pathways following infection. Similarly, the anti-pyroptotic activity of DAPs was entirely NRF2-dependent: in NRF2-deficient macrophages, DAPs neither reduced pyroptotic cell death (Figure 4L) nor decreased LDH (Figure 4M) synthesis. Furthermore, they failed to inhibit the maturation and secretion of IL-1β (Figure 4N) and IL-18 (Figure 4O), and did not suppress protein expression of key NLRP3 inflammasome components, including NLRP3, Caspase-1, and GSDMD-NT (Figure 4P–4S). Collectively, these findings establish NRF2 not only as the central regulator of DAPs’ antioxidant actions but also as an indispensable upstream node that integrates and coordinates DAPs-mediated suppression of inflammation and pyroptosis during *Salmonella* infection.

### Deer antler peptides inhibit *Salmonella* colonization and invasion in murine colonic tissues

To further investigate the functional role of NRF2 in mediating DAPs’ immunomodulatory effects during *Salmonella* infection *in vivo*, we established an acute colitis model through the oral gavage of *Salmonella* in WT and *nrf2*^−/−^ mice. As expected, both WT and *nrf2*^−/−^ mice exhibited significant weight change following bacterial challenge; however, DAPs pre-treatment had no effects on weight loss in either WT or *nrf2*^−/−^ mice (Figure 5A, 5B). Similarly, *Salmonella* infection induced pronounced colon shortening in both groups, while DAPs failed to preserve colon length in either genotype (Figure 5C–5E). In contrast, DAI scoring revealed a genotype-specific therapeutic response: DAPs significantly ameliorated clinical disease severity in WT mice (Figure 5F), whereas no improvement in DAI was observed in *nrf2*^−/−^ mice (Figure 5G), suggesting that NRF2 is essential for the *in vivo* anti-inflammatory and tissue-protective effects of DAPs. Notably, our quantitative analysis of bacterial burden in colonic homogenates demonstrated that DAPs pre-treatment significantly reduced *Salmonella* CFUs in both WT and *nrf2*^−/−^ mice (Figure 5H–5J), suggesting that the ability of DAPs to limit pathogen colonization is independent of NRF2. Crucially, our *in vitro* growth curve assays confirmed that DAPs (5 mg/mL) exerted no direct bacteriostatic or bactericidal activity against *Salmonella* (Supplement 2A), thereby ruling out intrinsic antimicrobial properties. Collectively, these findings indicate that the DAPs-mediated suppression of *Salmonella* burden *in vivo* is attributable to enhanced host-mediated clearance, potentially involving an NRF2-independent modulation of phagocyte function rather than a direct pathogen inhibition of bacterial growth.

### Deer antler peptides attenuate inflammation in *Salmonella*-infected mice in an NRF2-dependent manner

To investigate the modulatory role of DAPs in *Salmonella*-induced intestinal inflammation, we conducted comprehensive histopathological and molecular analyses of colonic tissues from infected mice. Histological scoring revealed that *Salmonella* infection triggered severe colonic inflammation in WT mice, characterized by goblet cell depletion, crypt loss, and inflammatory cell infiltration, whereas DAPs pretreatment significantly attenuated these pathological alterations (Figure 6A). In contrast, no significant histopathological improvement was observed in *nrf*2^−/−^ mice, indicating that the anti-inflammatory efficacy of DAPs is strictly dependent on a functional NRF2 signaling pathway. Consistent with these observations, ELISA quantification demonstrated that DAPs significantly reduced TNF-α and IL-6 protein levels in colonic tissue homogenates from infected WT mice, whereas this suppression was not seen in *nrf2*^−/−^ mice (Figure 6B–6E). Mechanistically, immunoblotting analysis confirmed that DAPs inhibited the infection-induced phosphorylation of ERK, JNK, and p38 signaling pathways in colonic lysates from WT mice (Figure 6F–6I); however, this inhibitory effect was entirely absent in the colonic tissues from *nrf2*^−/−^ mice (Figure 6J–6K). Collectively, these results demonstrate that DAPs alleviate *Salmonella*-driven colitis through an NRF2-dependent mechanism involving the coordinated suppression of MAPK pathway activation and downstream pro-inflammatory cytokine production.

### Deer antler peptides suppress *Salmonella*-induced oxidative stress via NRF2 signaling pathway

To define the essential role of NRF2 in DAPs-mediated antioxidant protection, we comprehensively evaluated its effects on oxidative stress biomarkers, antioxidant enzyme activity, and the expression of canonical downstream effector proteins in colonic tissues from *Salmonella*-infected mice. DAPs pre-treatment significantly suppressed *Salmonella*-induced COX-2 upregulation in WT mice, whereas this inhibition was entirely abrogated in *nrf2*^−/−^ mice (Figure 7A, 7B). Consistently, DAPs markedly attenuated lipid peroxidation in colonic tissues from WT mice, as evidenced by the significant reduction in MDA levels and concomitant restoration of GSH content. However, neither effect was observed in *nrf2*^−/−^ mice (Figure 7C–7F). Furthermore, immunoblotting analysis revealed that DAPs significantly enhanced the nuclear translocation of NRF2 and upregulated protein expression of its canonical downstream targets HO-1 and NQO-1 in colonic tissue lysates from WT mice (Figure 7G–7I). Critically, these molecular responses were entirely absent in the colonic tissues from *nrf2*^−/−^ mice (Figure 7G, 7K, 7L). Collectively, these findings demonstrate that DAPs exert antioxidant effects in *Salmonella*-induced colitis exclusively through an NRF2-dependent transcriptional activation of the endogenous antioxidant defense system.

### Deer antler peptides suppress *Salmonella*-induced pyroptosis via NRF2 signaling pathway

We have demonstrated that DAPs pre-treatment significantly suppressed *Salmonella*-induced pyroptosis in macrophages. To determine whether this anti-pyroptotic activity confers physiologically relevant protection i*n vivo* and whether it requires functional NRF2 signaling pathway, we assessed pyroptosis activation in colonic tissues from WT and *nrf2*^−/−^ mice infected orally with *Salmonella*. We found that DAPs significantly attenuated LDH synthesis in colonic tissue homogenates from infected WT mice (Figure 8A), whereas no significant reduction was observed in *nrf2*^−/−^ mice (Figure 8B). Consistently, DAPs significantly inhibited the proteolytic maturation and secretion of IL-1β and IL-18 in colonic tissue homogenates from WT mice (Figure 8C, 8E), whereas this suppression was not seen in *nrf2*^−/−^ mice (Figure 8D, 8F). Immunoblot analysis further demonstrated that DAPs significantly downregulated the protein expression of core NLRP3 inflammasome components, including NLRP3, Caspase-1, and GSDMD-NT in colonic tissue lysates from WT mice (Figure 8G–8J); meanwhile, none of these inhibitory effects were detectable in colonic tissues from *nrf2*^−/−^ mice (Figure 8K–8N). Collectively, these findings demonstrate that DAPs exert *in vivo* protection against *Salmonella*-induced colitis by selectively inhibiting NLRP3 inflammasome assembly and activation in an NRF2-dependent manner, thereby blocking downstream pyroptotic cell death and alleviating intestinal inflammation.

### Deer antler peptides maintain intestinal barrier integrity during *Salmonella* infection in an NRF2-dependent manner

LPS, a major structural component of the outer membrane of Gram-negative bacteria, induces damage in goat ruminal epithelial cells, and this injury can be mitigated by suppressing inflammatory responses and oxidative stress [26]. Additionally, DAPs suppress *Salmonella*-induced oxidative stress, inflammation, and pyroptosis in an NRF2-dependent manner, pathological processes that are known to act synergistically in disrupting intestinal epithelial barrier integrity. Therefore, we hypothesized that DAPs protect against infection-associated epithelial barrier dysfunction. To test this hypothesis, we first quantified intestinal permeability *in vivo* using the FITC-dextran assay. We found that *Salmonella* infection significantly increased serum FITC-dextran levels in WT mice, confirming severe barrier disruption, whereas pre-treatment with DAPs significantly attenuated this increase, indicating an effective preservation of epithelial barrier function (Figure 9A). In contrast, this protective effect was entirely absent in *nrf2*^−/−^ mice (Figure 9B), establishing NRF2 as an essential mediator of the barrier-stabilizing activity of DAPs. Mechanistically, immunohistochemical analysis revealed that DAPs significantly upregulated the protein expression of key epithelial barrier proteins, including MUC2, ZO-1, and Occludin in colonic tissues from infected WT mice (Figure 9C–9E); however, no such upregulation was observed in *nrf2*^−/−^ mice (Figure 9C–9E). Collectively, these findings demonstrate that DAPs maintain intestinal epithelial barrier structure and function by activating the NRF2 pathway to coordinately suppress oxidative stress, the inflammatory response, and the pyroptosis axis, whose integrated inhibition is required for sustained barrier homeostasis.

## DISCUSSION

*Salmonella* infection represents one of the most prevalent and economically impactful bacterial enteric diseases in livestock and poultry production systems [27]. The pathogen compromises intestinal epithelial barrier integrity, elicits robust pro-inflammatory responses, induces redox imbalance, and drives pyroptotic cell death, collectively impairing animal health, growth performance, and feed conversion efficiency [28]. Against the backdrop of growing global concerns regarding antimicrobial resistance, residual antibiotic contaminants in animal-derived food products, and antibiotic-associated gut microbiota dysbiosis, there is an urgent imperative to identify safe, efficacious, and sustainable alternatives for the prevention and control of enteric infections. DAPs, bioactive peptides isolated through the enzymatic hydrolysis and purification of deer antler velvet, have demonstrated potent anti-inflammatory, antioxidant, and immunomodulatory properties across diverse preclinical models [28]. Nevertheless, their efficacy against bacterial enteropathogens, particularly their capacity to mitigate *Salmonella*-induced intestinal inflammation and the molecular mechanisms underlying this process remains largely unknown. In this study, we systematically evaluate the protective effects of DAPs against *Salmonella*-induced intestinal injury using both *in vitro* and *in vivo* models, with mechanistic emphasis on the regulation of inflammatory signaling, pyroptosis, and oxidative stress.

DAPs exert potent anti-inflammatory effects through multiple coordinated mechanisms: these include the suppression of the pro-inflammatory enzymes COX-2 and 5-LOX; the downregulation of key cytokines TNF-α and IL-6 at both mRNA and protein levels; and the inhibition of central inflammatory signaling pathways, such as the MAPK signaling pathway, thereby attenuating the amplification and propagation of inflammatory cascades [29]. Accordingly, we first evaluated the anti-inflammatory efficacy of DAPs in *Salmonella*-infected murine macrophages. Consistent with prior reports, DAPs treatment significantly reduced TNF-α and IL-6 mRNA expression and protein secretion and suppressed the phosphorylation of ERK, JNK, and p38 signaling pathways, suggesting its functional modulation of canonical pro-inflammatory signaling axes. Notably, inflammation and pyroptosis are mechanistically interlinked: pathogen-associated molecular patterns, such as LPS prime NLRP3 inflammasome assembly, while GSDMD-mediated pore formation executes pyroptosis, facilitating the maturation and extracellular release of IL-1β and IL-18, along with DAMPs, thereby perpetuating secondary inflammatory responses [30]. Given DAPs’ robust suppression of upstream inflammatory drivers, we hypothesized that they may concurrently inhibit pyroptotic cell death. Supporting this, DAPs markedly decreased pyroptosis in *Salmonella*-infected macrophages. Collectively, these findings demonstrate that DAPs confer a dual inhibition of inflammation and pyroptosis in *Salmonella*-challenged macrophages, highlighting their potential as a multi-target therapeutic agent for enteric bacterial infection.

NRF2, the master transcriptional regulator of cellular antioxidant defense and a pivotal modulator of inflammatory responses, serves as a central molecular target mediating the anti-inflammatory effects of DAPs [31]. Accumulating evidence has demonstrated that DAPs activate the NRF2 signaling pathway, leading to a coordinated upregulation of downstream cytoprotective genes, including HO-1 and NQO-1, thereby restoring redox homeostasis and suppressing excessive inflammatory responses [23]. These findings are consistent with the results of our study that DAPs treatment significantly suppressed oxidative stress in *Salmonella-*infected macrophages predominantly through a canonical activation of the NRF2 pathway. Notably, in NRF2-deficient macrophages, DAPs completely failed to suppress *Salmonella*-induced oxidative stress. Critically, in the absence of NRF2, DAPs failed to inhibit inflammatory responses and pyroptosis in *Salmonella*-infected macrophages. Collectively, these findings demonstrate that DAPs confer integrated protection against *Salmonella*-induced immunopathology by activating the NRF2 signaling axis to suppress MAPK-driven inflammatory signaling and NLRP3/Caspase-1/GSDMD-mediated pyroptosis. However, whether DAPs activate NRF2 through direct molecular engagement or indirectly via a modulation of upstream signaling pathways remains to be determined.

To further define the functional contribution of NRF2 to DAPs-mediated protection against *Salmonella*–induced intestinal inflammatory injury, we established an acute colitis model in WT and *nrf2*^−/−^ mice via oral gavage with *Salmonella*. Notably, DAPs reduced *Salmonella* loads in the colonic tissues of both WT and *nrf2*^−/−^ mice. Crucially, *in vitro* antimicrobial assays confirmed that DAPs had no direct bacteriostatic or bactericidal activity against *Salmonella*, indicating that their *in vivo* antibacterial effect may be associated with enhanced macrophage phagocytosis, upregulated antimicrobial peptides, or a modulated inflammatory microenvironment to restrict bacterial niche establishment and replication, though additional experiments are needed to confirm this. To date, no published studies have demonstrated that DAPs exert regulatory effects on the growth of pathogenic bacteria. In contrast, a single study has provided preliminary evidence that pilose antler polypeptides may modulate the composition of the intestinal microbiota [22]. Further histopathological and molecular analyses revealed that DAPs significantly attenuated *Salmonella*-induced colonic tissue inflammatory response in WT mice, but had no effects on *nrf2*^−/−^ mice, thereby establishing NRF2 as indispensable for DAPs’ anti-inflammatory and tissue-protective actions *in vivo*. These findings are consistent with previous studies demonstrating that DAPs alleviate acetaminophen-induced oxidative stress and apoptosis by activating the NRF2/Keap1/HO-1 pathway [32], and that DAPs exert neuroprotective effects in a Parkinson’s disease model through a modulation of oxidative damage via the AKT/NRF2/HO-1 axis [33]. Moreover, DAPs robustly inhibited pyroptosis in the colonic tissues of *Salmonella*-infected WT mice, but this anti-pyroptotic effect was not seen in *nrf2*^−/−^ mice. Similarly, DAPs effectively mitigated oxidative stress in WT colonic tissue, whereas they failed to normalize redox parameters in *nrf2*^−/−^ mice. This finding is consistent with a prior study demonstrating that sika deer antler protein mitigates acetaminophen-induced oxidative stress through an activation of the NRF2/Keap1/HO-1 signaling pathway [32]. Collectively, our data identify NRF2 as a central and non-redundant signaling hub through which DAPs coordinately suppress oxidative stress, inflammation, and pyroptosis during *Salmonella*-induced colitis. Intriguingly, although pyroptosis is conventionally regarded as host-protective, primarily by facilitating pathogen clearance, our results reveal a more nuanced paradigm: DAPs achieve potent antibacterial efficacy against *Salmonella* despite suppressing pyroptosis, and this bactericidal effect is preserved in *nrf2*^−/−^ mice. These observations suggest that DAPs engage complementary, NRF2-independent immune regulatory mechanisms, such as the enhancement of macrophage phagocytic function or the potentiation of autophagy-mediated intracellular pathogen degradation to eliminate *Salmonella* without a dependence on inflammatory cell lysis.

In this study, we have demonstrated that DAPs exert comprehensive protection of the intestinal epithelial barrier during *Salmonella* infection predominantly through an activation of the NRF2 signaling pathway. Inflammation, oxidative stress, and pyroptosis are associated with increased intestinal paracellular permeability [34]. *Salmonella* infection has been consistently demonstrated to increase intestinal permeability and compromise intestinal barrier integrity, whereas DAPs significantly attenuated *Salmonella*-induced intestinal permeability in WT mice, as evidenced by the systemic levels of FITC-dextran, and preserved the expression of critical barrier effectors, including the secreted mucin MUC2, and the transmembrane tight junction proteins Claudin-1 and ZO-1. This finding is consistent with a prior study showing that DAPs significantly attenuate DSS-induced colonic inflammation in mice; suppress the secretion of pro-inflammatory cytokines TNF-α, IL-6, and IL-1β; and thereby maintains intestinal barrier integrity [35]. However, all *in vivo* experimental results were obtained exclusively in male mice; therefore, further studies including both sexes of mice are required to fully define the role of DAPs in *Salmonella* infection.

## CONCLUSION

Our findings demonstrate that DAPs activate NRF2 and subsequently suppress *Salmonella* infection-induced inflammation, oxidative stress, and pyroptosis, as illustrated in Figure 10. Collectively, this work not only expands the functional repertoire of DAPs beyond their classical role as an antioxidant but also establishes a paradigm for host-directed, multi-targeted antimicrobial strategies that harness natural animal-derived peptides to combat Gram-negative enteric pathogens. However, DAPs are a complex, naturally derived peptide mixture containing multiple structurally and functionally distinct bioactive constituents. The precise constituent(s) responsible for NRF2 activation, which thereby drive the immunomodulatory effects observed during *Salmonella* infection have not yet been delineated. Elucidating these components represents a central objective of our ongoing and future mechanistic investigations.

## Figures and Tables

**Figure 1:**
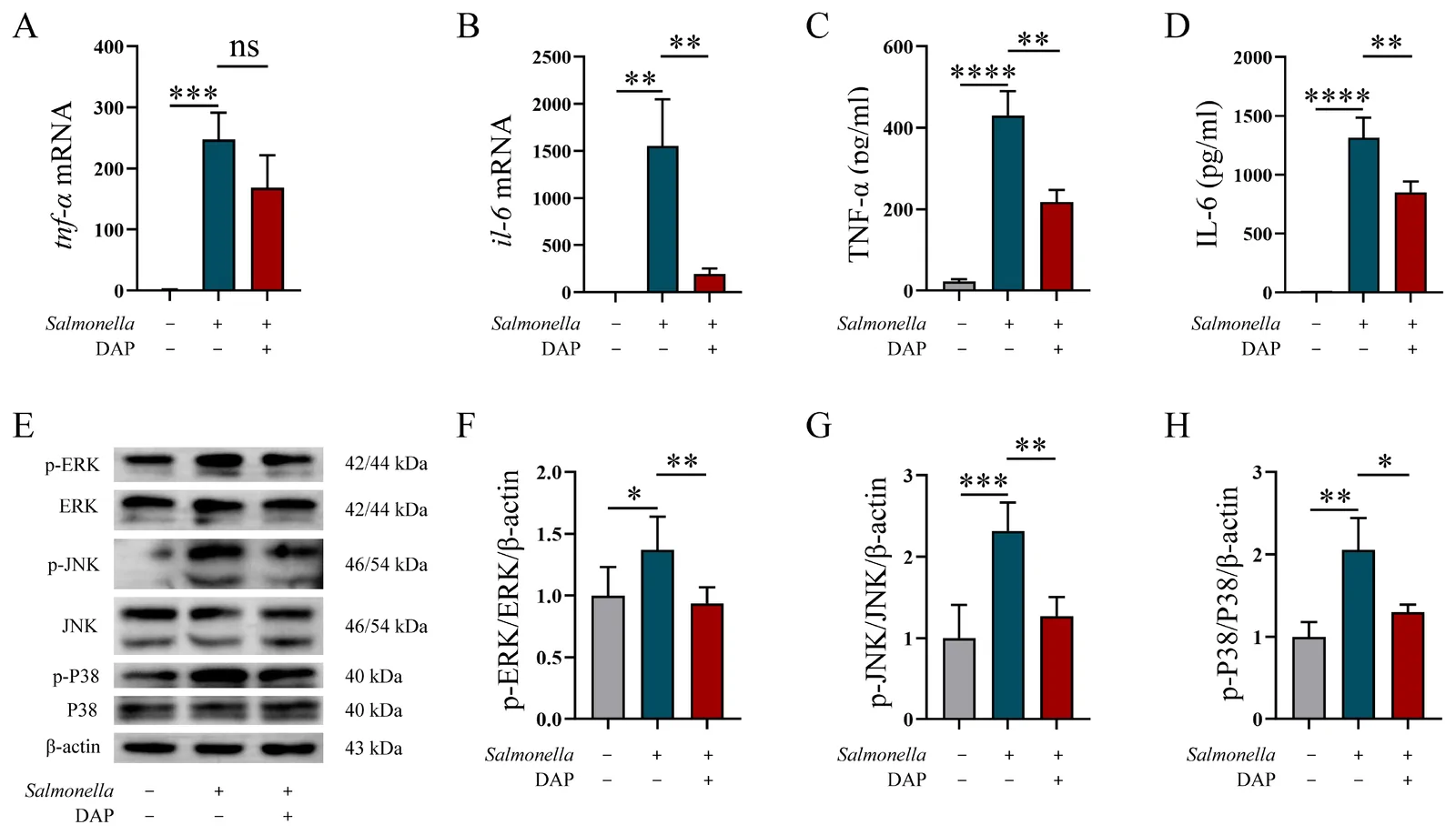
DAPs inhibit inflammatory response during *Salmonella* infection. (A, B) qRT-PCR was used to assess the expression level of TNF-α, and IL-6 mRNA. (C, D) ELISA was used to analyze the content of TNF-α, and IL-6 protein in the culture medium supernatant of macrophages. (E–H) Western blot analyzed the phosphorylation levels of ERK (E, F), JNK (E, G), P38 (E, H) in *Salmonella*-infected macrophages. Data shown as means±SD, * p<0.05, ** p<0.01, *** p<0.001, and **** p<0.0001. ns, not significant; DAP, deer antler peptide; qRT-PCR, quantitative reverse transcription polymerase chain reaction; ELISA, enzyme-linked immunosorbent assay; SD, standard deviation.

**Figure 2:**
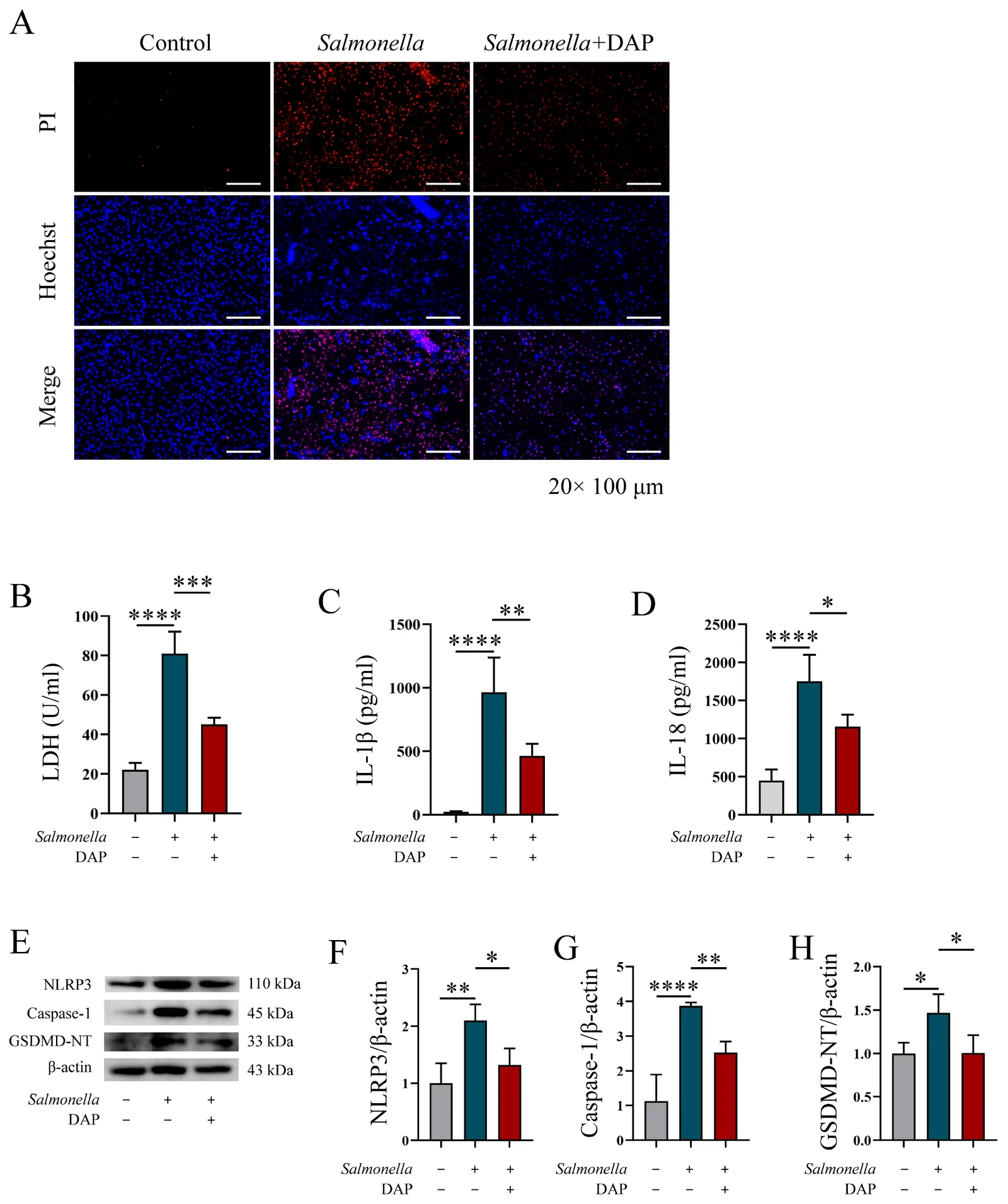
DAPs mitigate pyroptosis in Salmonella-infected macrophages. (A) Macrophages were infected with *Salmonella* and treated with DAPs, followed by Hoechst 33342 (blue, nuclear staining) and PI (red, membrane integrity indicator) double staining. The images were 20× magnification, and scale bar is 100 μm. (B) LDH synthesis in the culture supernatant was quantified using a commercial assay kit according to the manufacturer’s instructions. (C, D) Levels of IL-1β (C) and IL-18 (D) secreted in the macrophage culture medium were measured by ELISA. (E–G) Protein expression of NLRP3, Caspase-1, and GSDMD-NT in *Salmonella*-infected macrophages was assessed by Western blot. Data shown as means±SD, * p<0.05, ** p<0.01, *** p<0.001, and **** p<0.0001. DAP, deer antler peptide; PI, propidium iodide; ELISA, enzyme-linked immunosorbent assay; SD, standard deviation.

**Figure 3:**
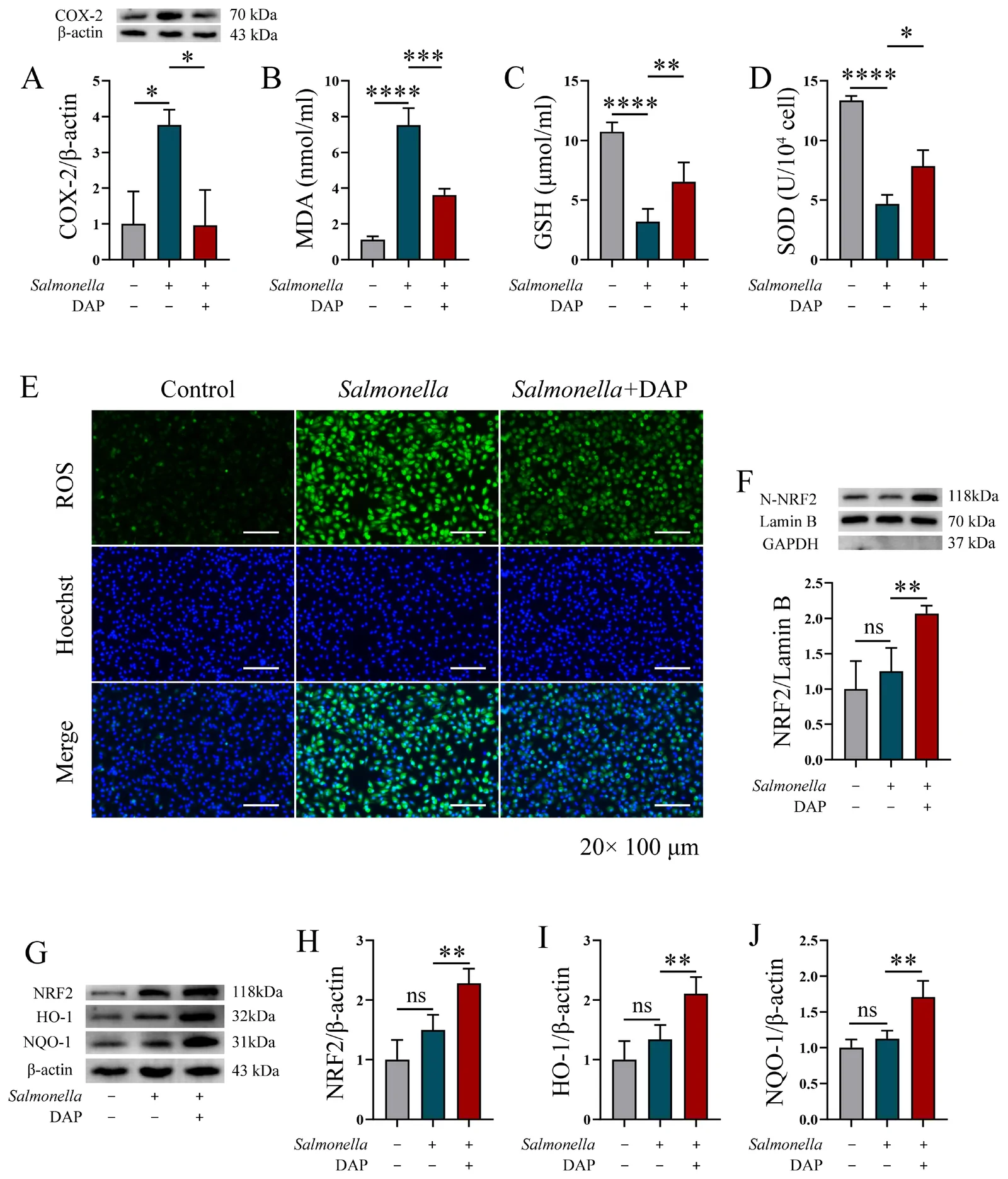
DAPs suppress oxidative stress in *Salmonella*-infected macrophages. (A) Protein expression of COX-2 was detected by Western blot. (B–D) The content of MDA and GSH, as well as the enzymatic activity of SOD, were measured in *Salmonella*-infected macrophages using commercial assay kits. (E) Macrophages were infected with *Salmonella*, followed by Hoechst 33342 (blue, nuclear staining) and DCFH-DA (green, ROS) double staining. The images were 20× magnification; scale bar is 100 μm. (F) Nuclear translocation of NRF2 in macrophages was assessed by Western blot. (G–J) Protein levels of NRF2, HO-1, and NQO-1 in *Salmonella*-infected macrophages were analyzed by Western blot. Data are presented as mean±SD. * p<0.05, ** p<0.01, *** p<0.001, **** p<0.0001. DAP, deer antler peptide; MDA, malondialdehyde; GSH, glutathione; SOD, superoxide dismutase; ROS, reactive oxygen species; ns, not significant; SD, standard deviation.

**Figure 4:**
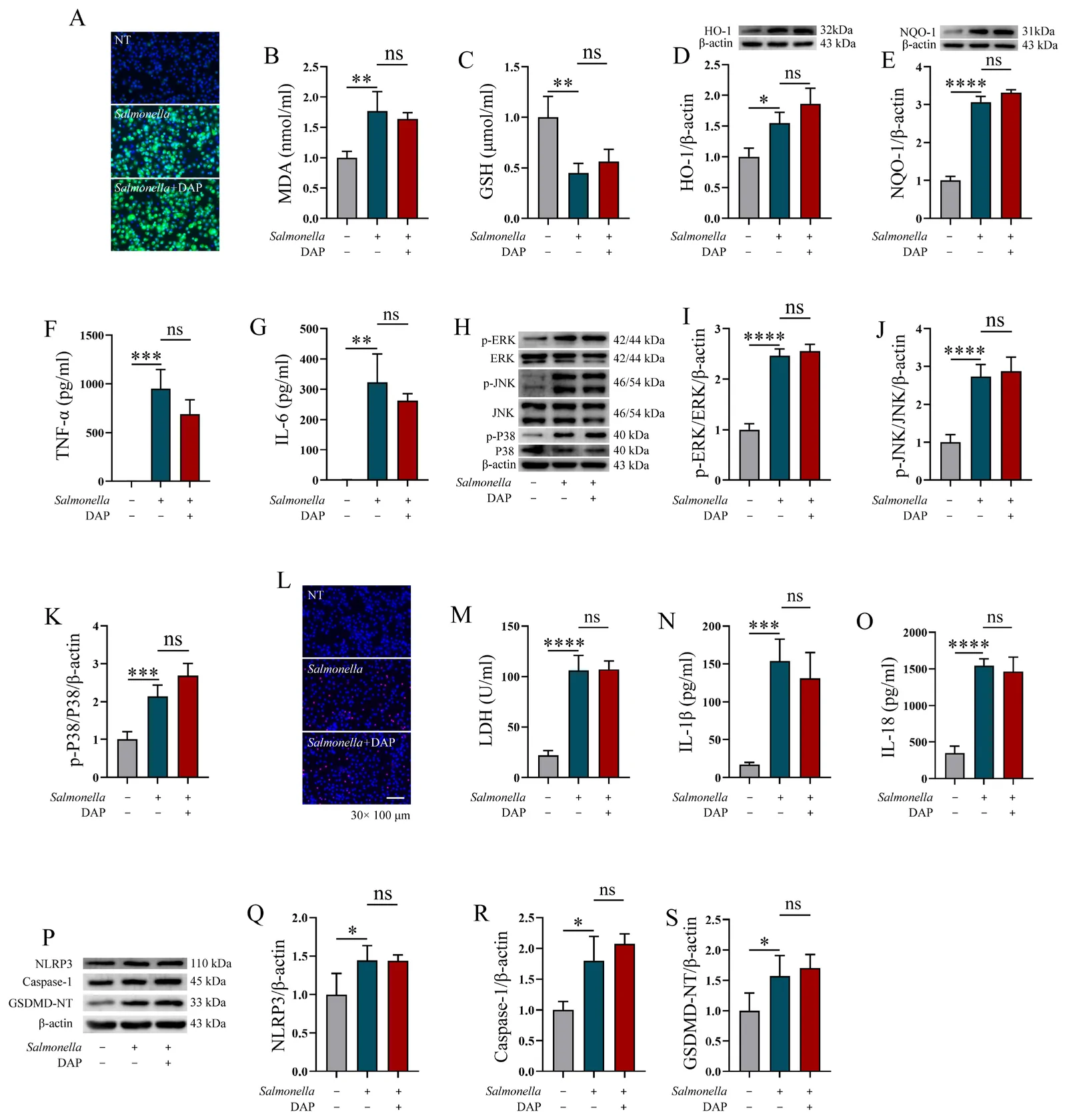
DAPs attenuate oxidative stress, inflammatory responses, and pyroptosis through activation of the NRF2 signaling pathway. (A) Macrophages were infected with *Salmonella* and subjected to Hoechst 33342 (blue, nuclear staining) and DCFH-DA (green, ROS indicator) double staining. The images were 20× magnification, scale bar is 100 μm. (B, C) The content of MDA and GSH, were measured in *Salmonella*-infected macrophages using commercial assay kits. (D, E) Protein levels of NRF2, HO-1, and NQO-1 in *Salmonella*-infected macrophages were analyzed by Western blot. (F, G) The concentrations of TNF-α and IL-6 in the macrophage culture supernatant were quantified by ELISA. (H–K) Phosphorylation levels of ERK (H, I), JNK (H, J), and p38 (H, K) in *Salmonella*-infected macrophages were assessed by Western blot. (L) Macrophages were infected with *Salmonella*, followed by Hoechst 33342 (blue, nuclear staining) and propidium iodide (PI, red, membrane integrity indicator) double staining. The images were 20× magnification, scale bar is 100 μm. (M) LDH synthesis in the culture supernatant was quantified using a commercial assay kit according to the manufacturer’s instructions. (N, O) Levels of IL-1β and IL-18 secreted into the macrophage culture medium were measured by ELISA. (P–S) Protein expression of NLRP3, Caspase-1, and GSDMD-NT in *Salmonella*-infected macrophages was assessed by Western blot. Data are presented as mean±SD, * p<0.05, ** p<0.01, *** p<0.001, and **** p<0.0001. DAP, deer antler peptide; ns, not significant; MDA, malondialdehyde; GSH, glutathione; ROS, reactive oxygen species; ELISA, enzyme-linked immunosorbent assay; SD, standard deviation.

**Figure 5:**
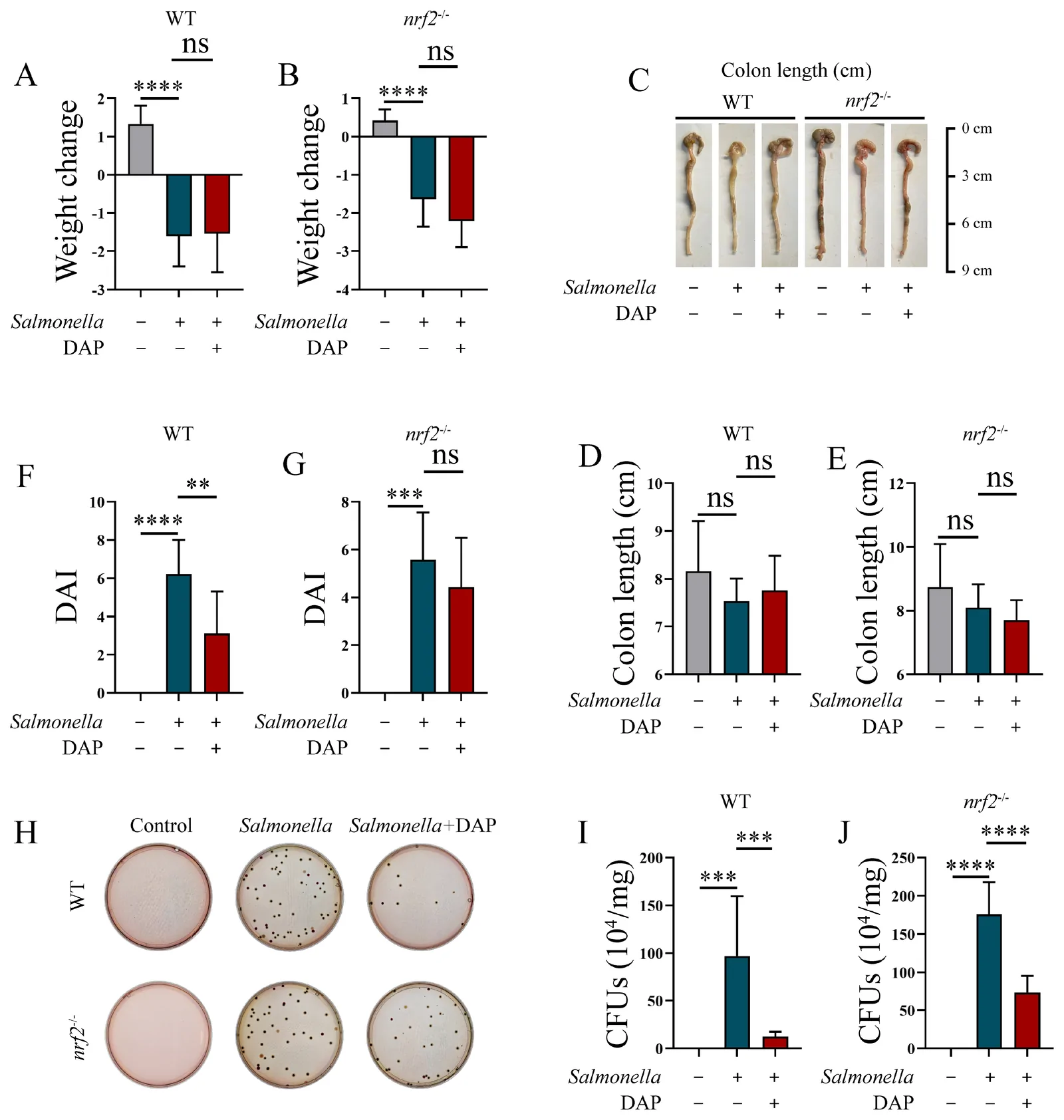
DAPs inhibit *Salmonella* colonization and invasion in the murine colonic tissues. (A, B) Body weight was recorded as W1 before the experiment and W2 after the experiment, with weight change calculated as W2–W1. (C–E) Colon lengths of WT and *nrf2*^−/−^ mice following *Salmonella* infection. (F, G) Disease activity index of WT and *nrf2*^−/−^ mice following *Salmonella* infection. (H–J) Colonic tissue homogenates from WT and *nrf2*^−/−^ mice were coated onto bacterial culture plates, incubated at 37°C for 24 h, and CFU were enumerated. Data are presented as means±SD, ** p<0.01, *** p<0.001, and **** p<0.0001. WT, wild-type; ns, not significant; DAP, deer antler peptide; SD, standard deviation.

**Figure 6:**
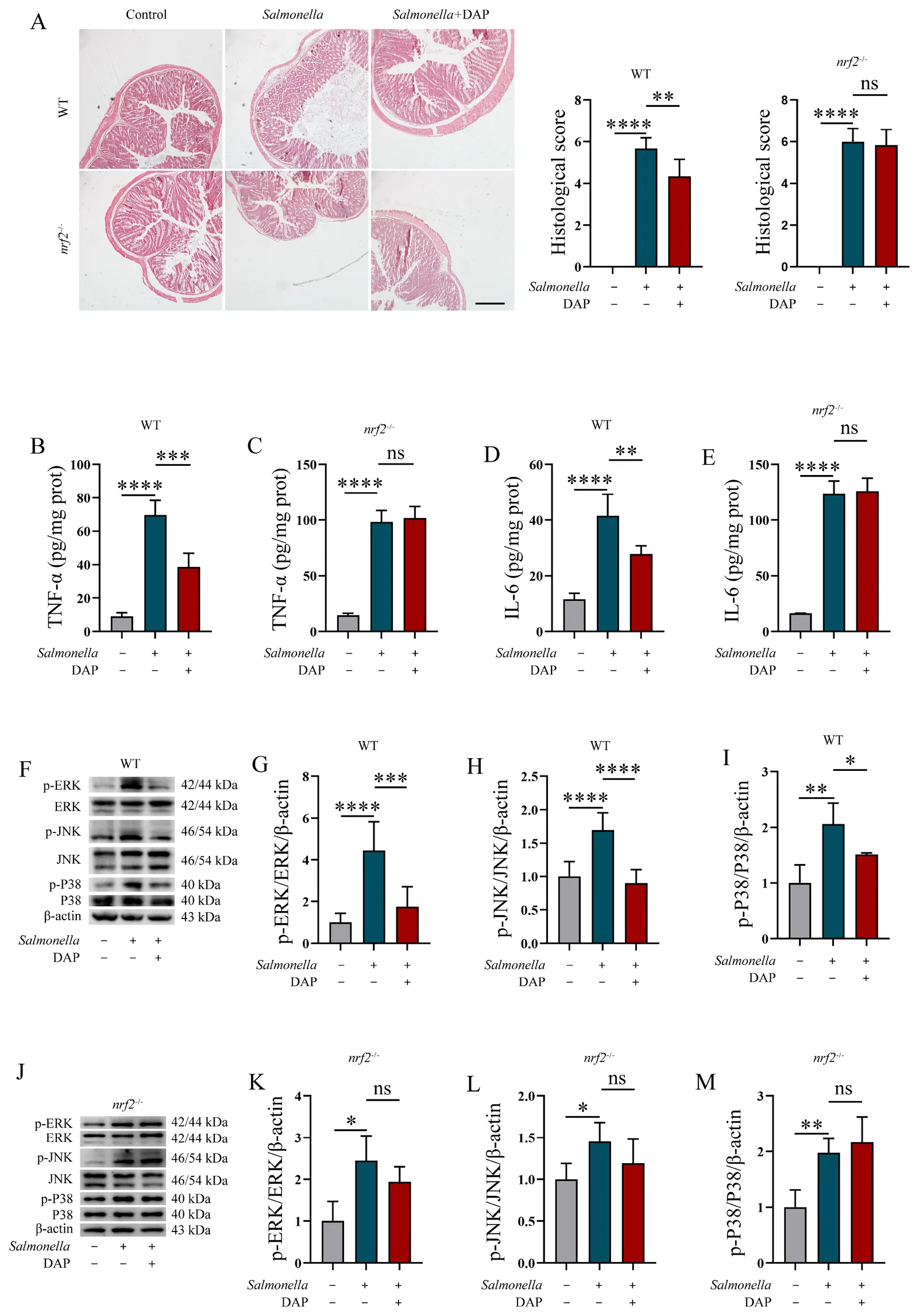
DAPs attenuate inflammation in *Salmonella*-infected mice in an NRF2-dependent manner. (A) H&E staining was performed to assess colonic tissue damage in *Salmonella*-infected WT and *nrf2*^−/−^ mice. The image was magnified 5×, with a scale bar representing 200 μm. (B–E) ELISA was used to measure TNF and IL-6 secretion in colonic tissues of *Salmonella*-infected WT and *nrf2*^−/−^ mice. (F–I) Western blot was conducted to analyze phosphorylation of ERK, JNK, and P38 signaling pathways in colonic tissues from WT mice. (J–M) Western blot was performed to assess the phosphorylation of ERK, JNK, and P38 signaling pathways in colonic tissue from *nrf2*^−/−^ mice. Data are presented as means±SD, * p<0.05, ** p<0.01, *** p<0.001, and **** p<0.0001. DAP, deer antler peptide; WT, wild-type; ns, not significant; H&E, hematoxylin and eosin; ELISA, enzyme-linked immunosorbent assay; SD, standard deviation.

**Figure 7:**
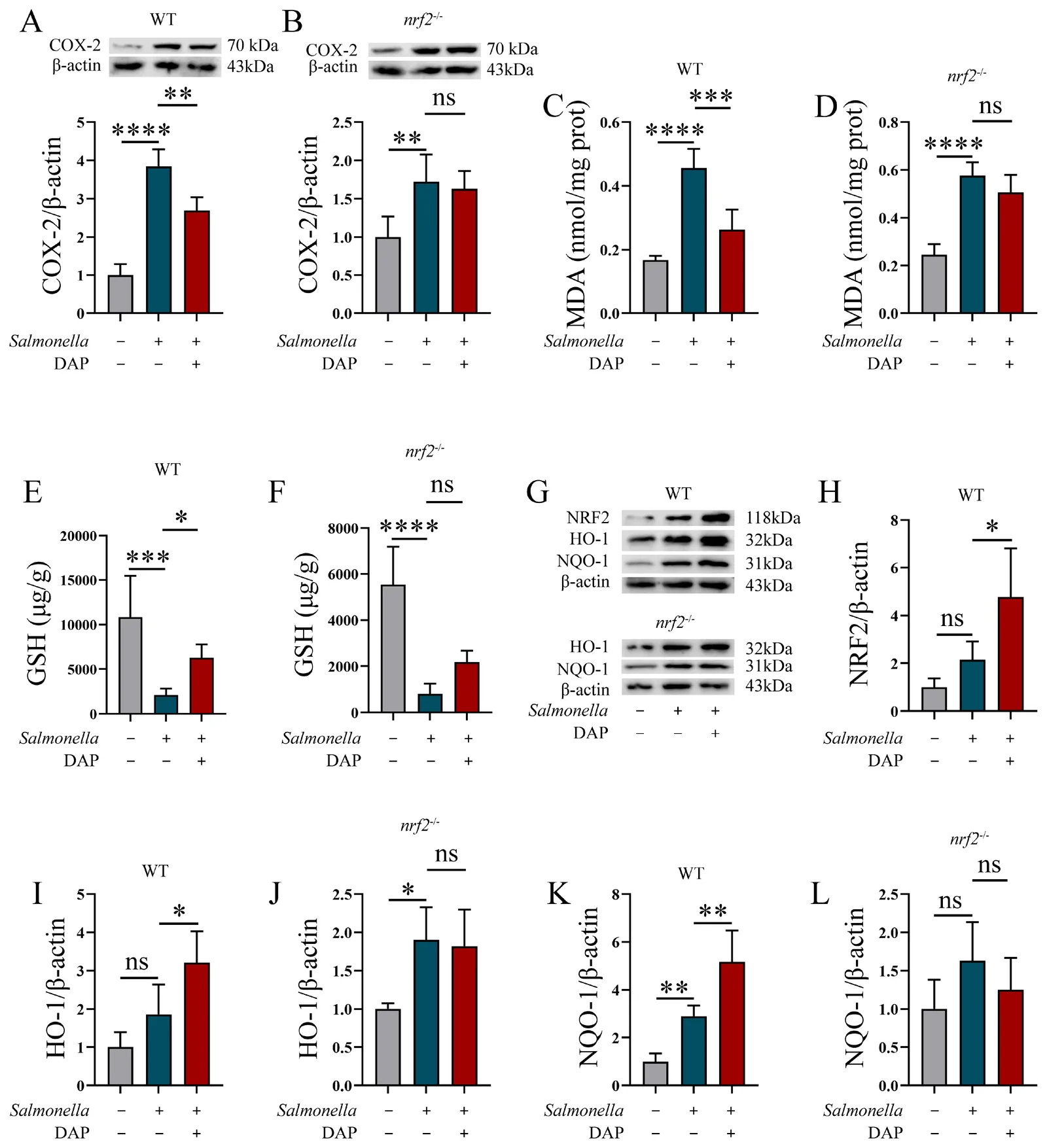
DAPs suppress *Salmonella*-induced oxidative stress via NRF2 signaling pathway. (A, B) Western blot was conducted to analyze COX-2 expression levels in colonic tissues from WT mice. (C–F) Commercial MDA and GSH assay kits were used to measure MDA and GSH contents in colonic tissues of WT and *nrf2*^−/−^ mice. (G–J) Western blot was performed to assess expression levels of NRF2, HO-1, and NQO-1 in colonic tissues from WT mice. (G, K, L) Western blot was conducted to evaluate expression levels of HO-1, and NQO-1 in colonic tissue from *nrf2*^−/−^ mice. Data are presented as means±SD, * p<0.05, ** p<0.01, *** p<0.001, and **** p<0.0001. WT, wild-type; DAP, deer antler peptide; ns, not significant; MDA, malondialdehyde; GSH, glutathione; SD, standard deviation.

**Figure 8:**
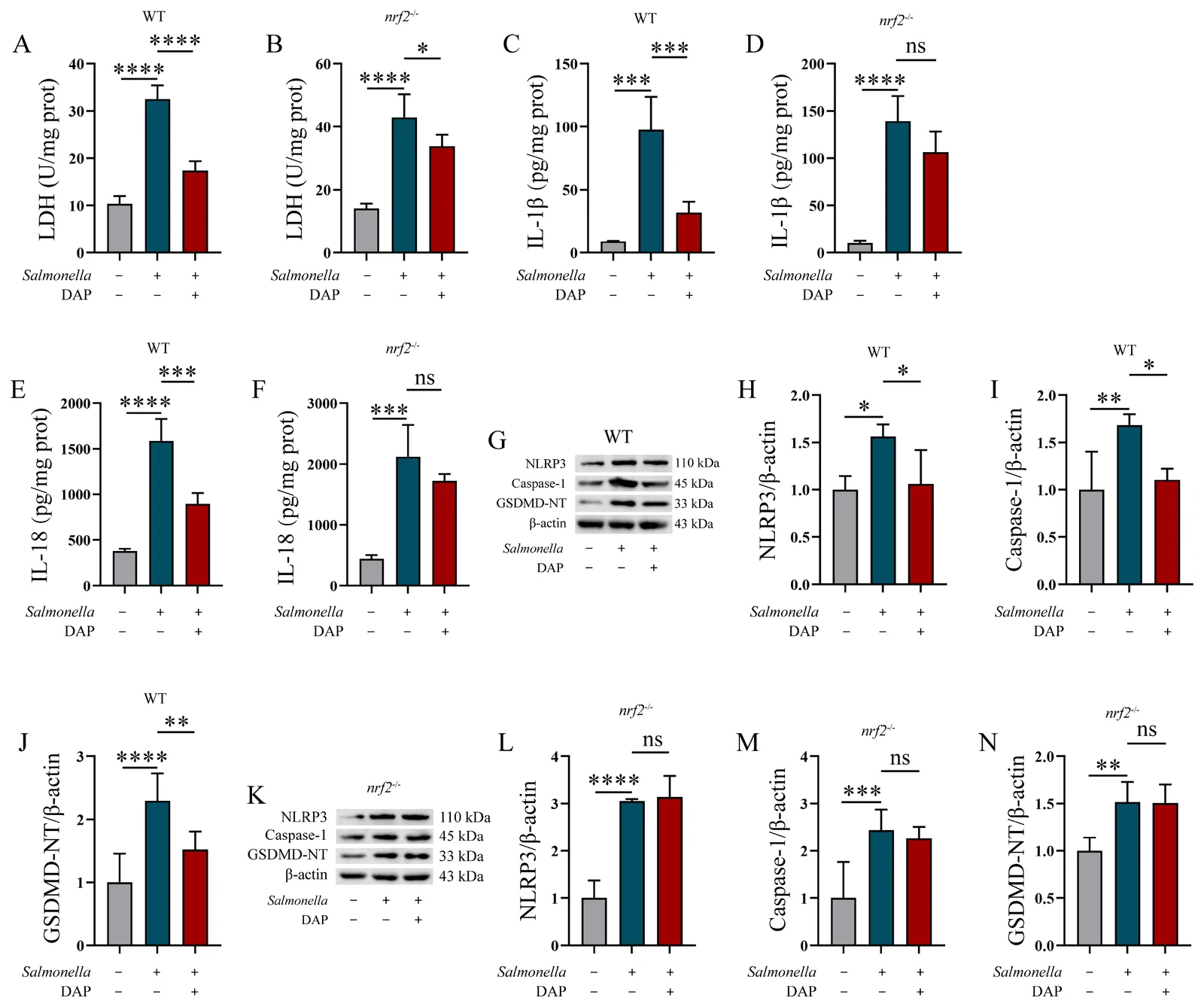
DAPs suppress *Salmonella*-induced pyroptosis via NRF2 signaling pathway. (A, B) A commercial LDH assay kit was used to measure LDH synthesis in colonic tissues of WT and *nrf2*^−/−^ mice. (C–F) Commercial ELISA kits were used to quantify IL-1β and IL-18 levels in colonic tissues from WT and *nrf2*^−/−^ mice. (G–J) Western blot was conducted to evaluate expression levels of NLRP3, Caspase-1, and GSDMD-NT in colonic tissues from WT mice. (K–N) Western blot was performed to assess expression levels of NLRP3, Caspase-1, and GSDMD-NT in colonic tissue from *nrf2*^−/−^ mice. Data are presented as means±SD, * p<0.05, ** p<0.01, *** p<0.001, and **** p<0.0001. WT, wild-type; DAP, deer antler peptide; ns, not significant; ELISA, enzyme-linked immunosorbent assay; SD, standard deviation.

**Figure 9:**
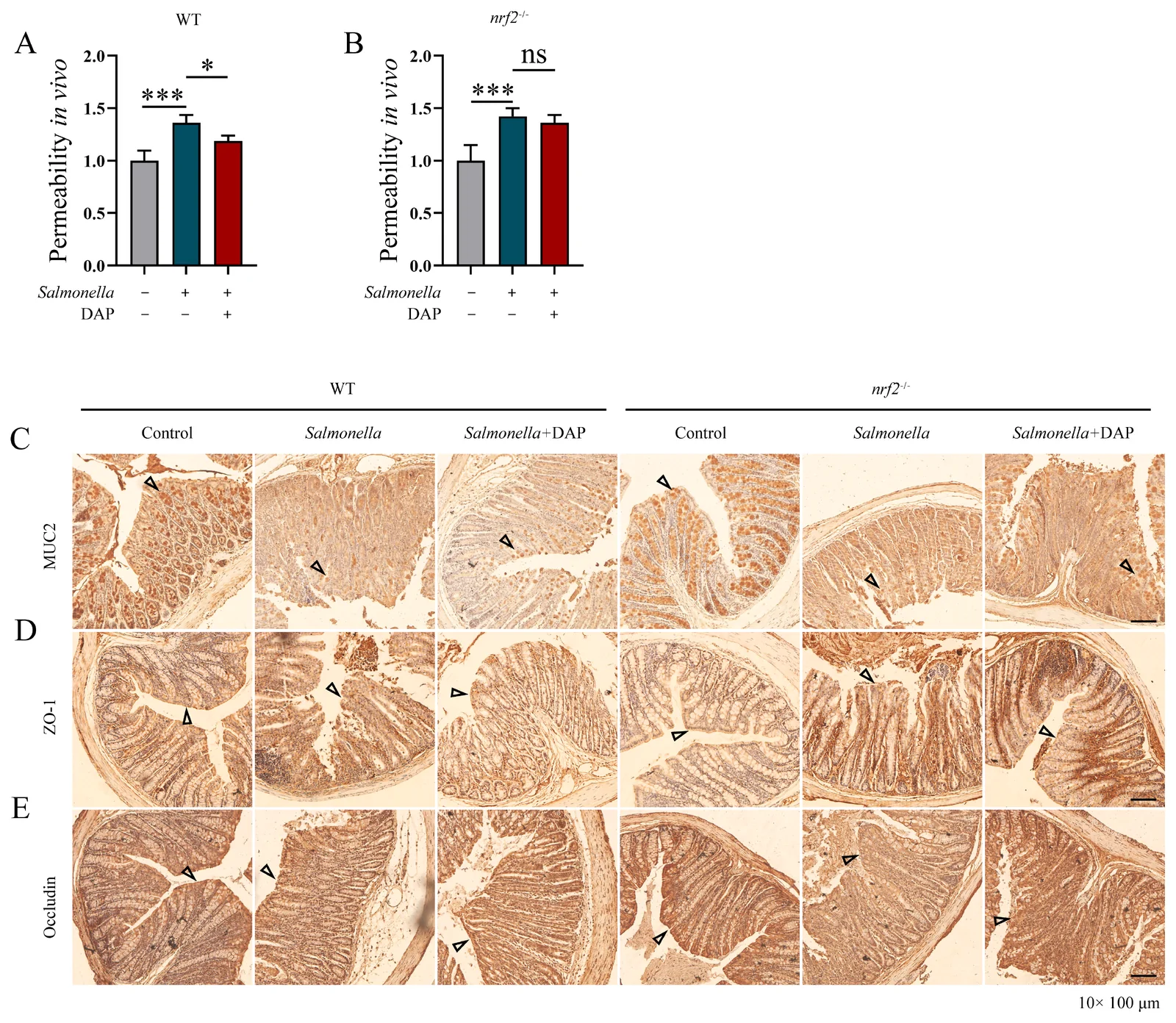
DAPs maintain intestinal barrier integrity during *Salmonella* infection in an NRF2-dependent manner. (A, B) FITC-dextran assay was performed to assess intestinal permeability in *Salmonella*-infected WT and *nrf2*^−/−^ mice. (C–E) Immunohistochemical staining was conducted to examine the expression of MUC2, ZO-1, and Occludin in colon tissues from WT and *nrf2*^−/−^ mice. The image was magnified 10×, with a scale bar representing 100 μm. The black arrow marks the subcellular localization of the target protein. Data are presented as means±SD, * p<0.05, and *** p<0.001. WT, wild-type; DAP, deer antler peptide; ns, not significant; SD, standard deviation.

**Figure 10:**
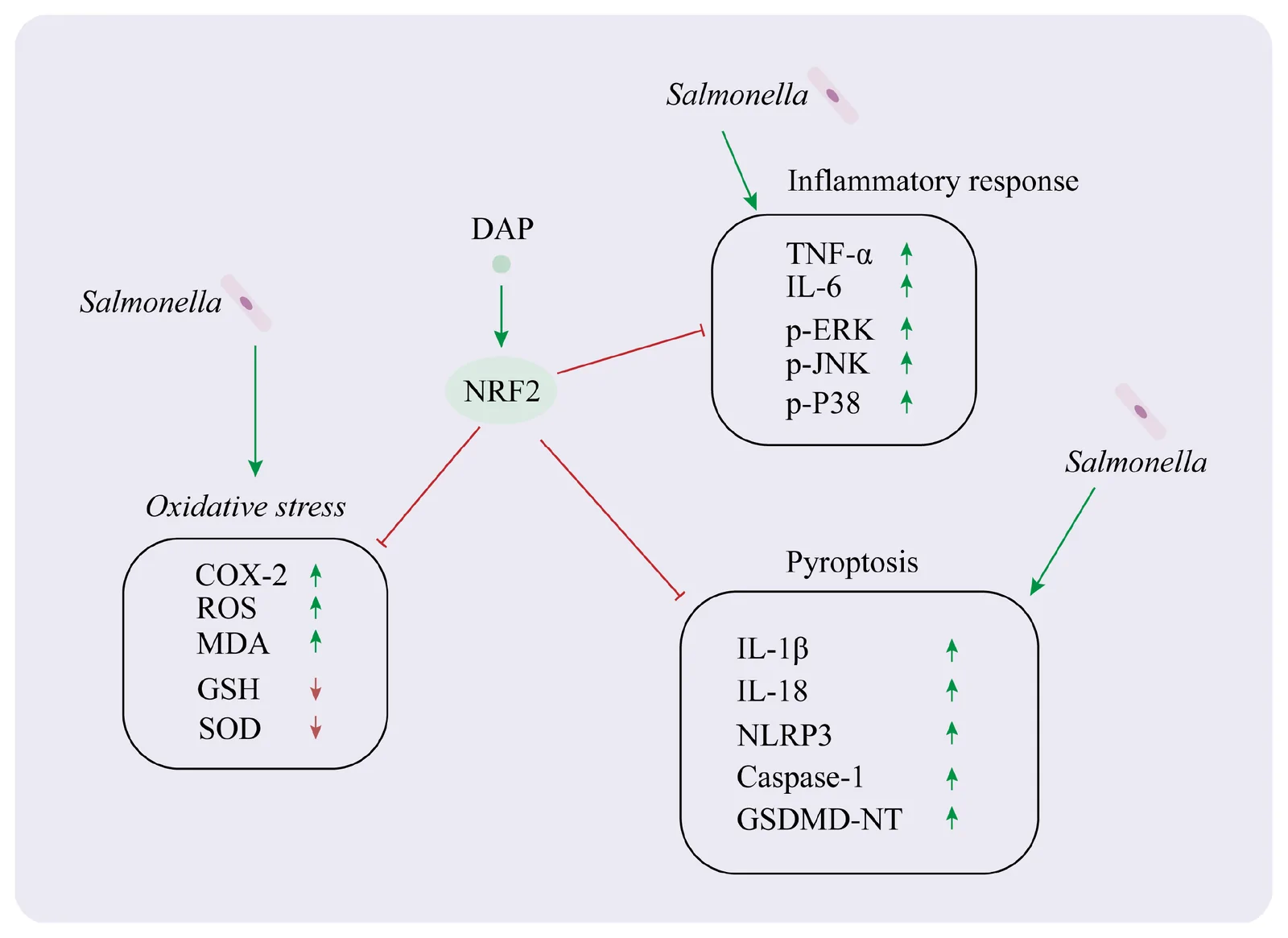
DAPs suppress *Salmonella*-induced inflammation, oxidative stress, and pyroptosis through NRF2 activation. DAP, deer antler peptide; ROS, reactive oxygen species; MDA, malondialdehyde; GSH, glutathione; SOD, superoxide dismutase.

## Data Availability

Upon reasonable request, the datasets of this study can be available from the corresponding author.

## SUPPLEMENTARY MATERIAL

**Supplement 1**. The effects of DAPs on the viability of macrophages. **Supplement 2**. The effects of DAPs on the growth curve of *Salmonella*.
