# Supplementary material for: Deer antler peptides attenuate inflammation, oxidative stress, and pyroptosis in Salmonella-infected macrophages and murine colonic tissues
Source: Anim Biosci. 2026 Sep 1;39(11):260612. doi: 10.5713/ab.260612 (PMC13740916; doi:10.5713/ab.260612)
Supplement: Supplementary file 1 [file ab-260612-Supplementary.pdf]

Supplement 1. The effects of DAPs on the viability of macrophages

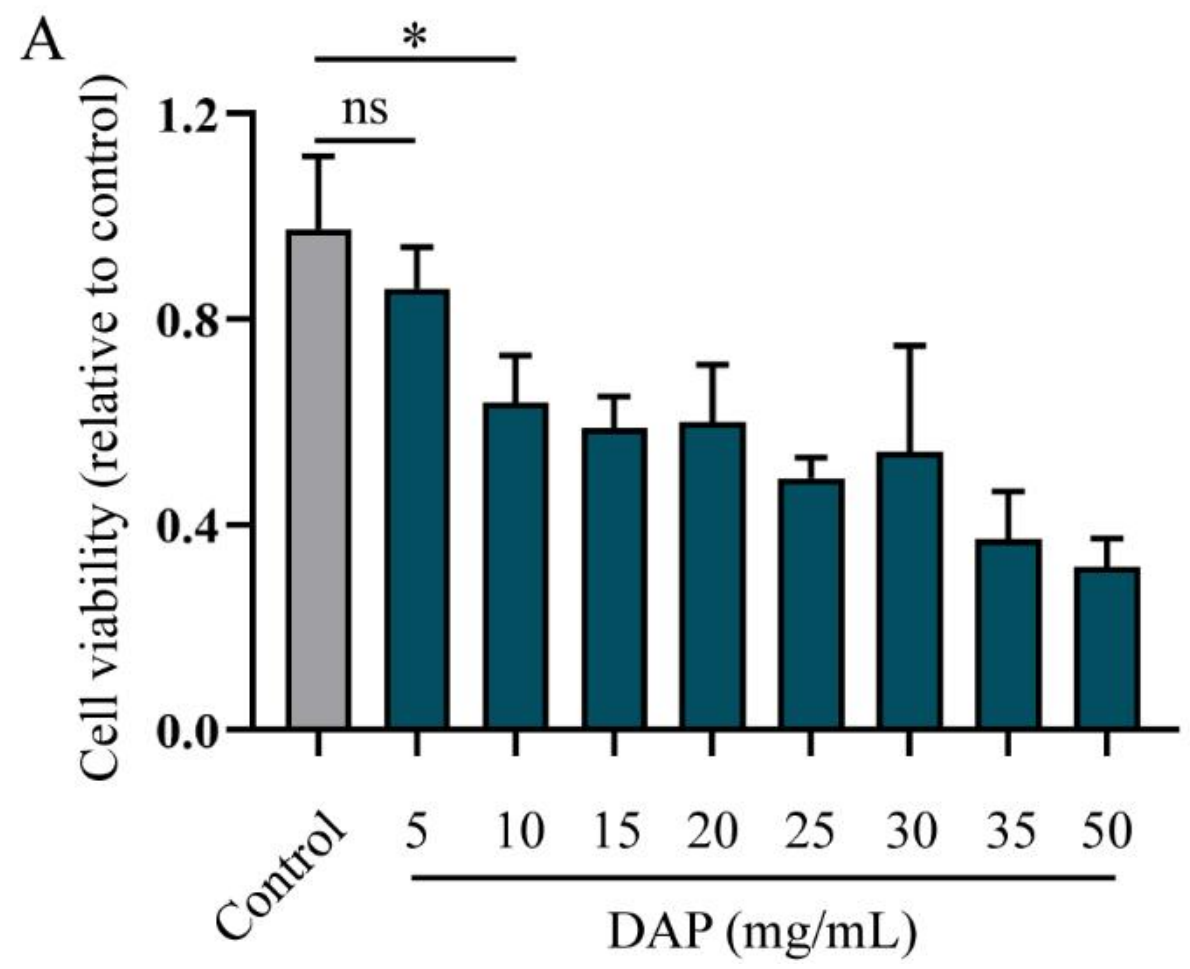

Supplement 1. (**A**) The effects of DAPs (5, 10, 15, 20, 25, 30, 35, and 50 mg/mL) on the viability of macrophages isolated from WT mice.

Supplement 2. The effects of DAPs on the growth curve of *Salmonella*

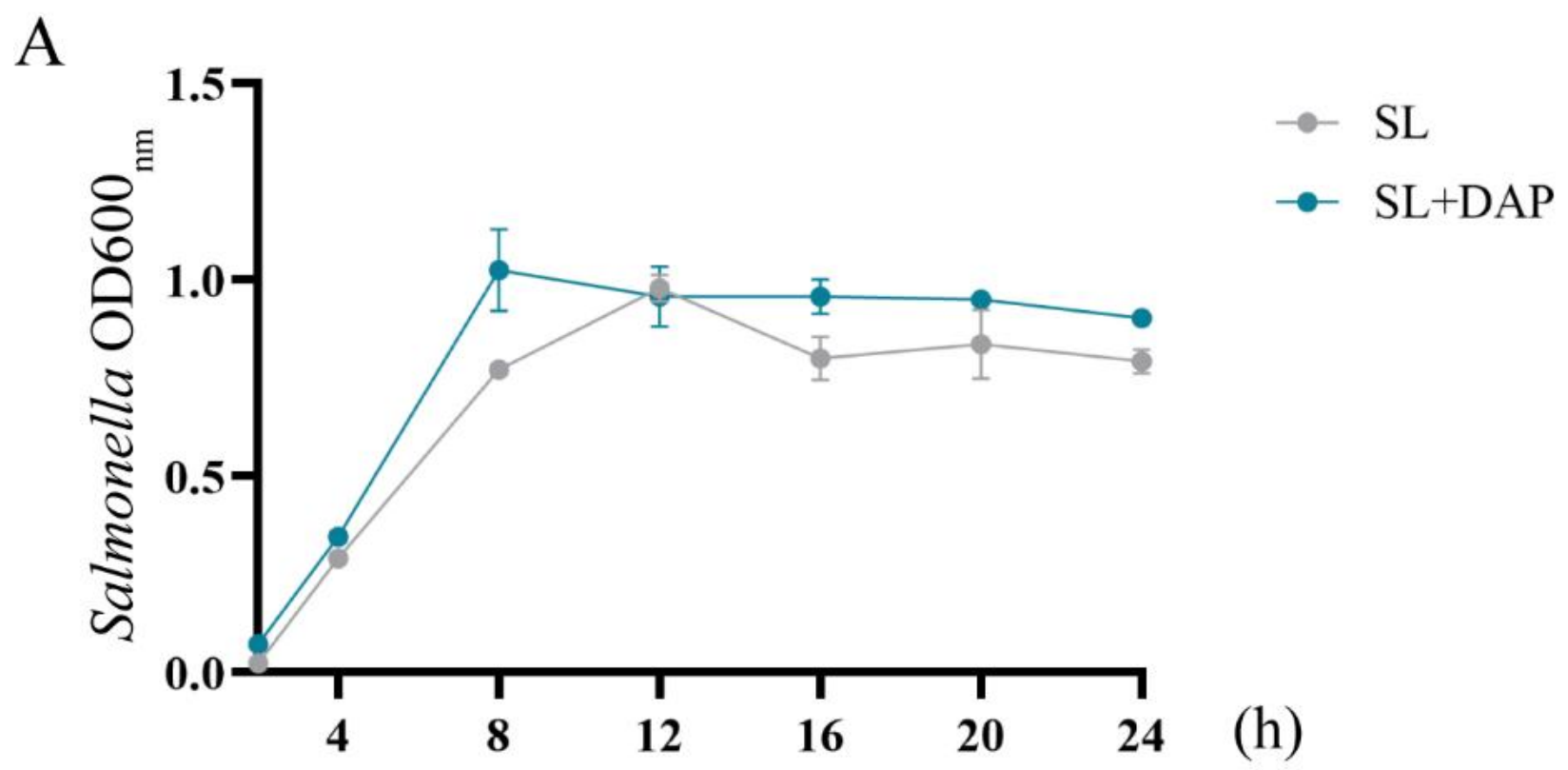

Supplement 2. (**A**) The growth curve of *Salmonella* cultured with DAPs (5 mg/mL) or without for 24 h.
